# Exploring a Possible Link between the Fecal Microbiota and the Production Performance of Pigs

**DOI:** 10.3390/vetsci9100527

**Published:** 2022-09-27

**Authors:** Yanan Cao, Fei Wang, Haifei Wang, Shenglong Wu, Wenbin Bao

**Affiliations:** 1 Key Laboratory for Animal Genetics, Breeding, Reproduction and Molecular Design, College of Animal Science and Technology, Yangzhou University, Yangzhou 225009, China; 2 Joint International Research Laboratory of Agriculture & Agri-Product Safety, Yangzhou University, Yangzhou 225009, China

**Keywords:** pigs, fecal microbiota, backfat thickness, *Lactobacillus reuteri*

## Abstract

**Simple Summary:**

The aim of this study is to investigate the relationship between fecal microbiota and the backfat thickness of pigs by 16S rRNA gene sequencing. Our study found that there were significant differences in the composition of microbiota at the species level, characterized by a higher abundance of *Lactobacillus reuteri* (*L. reuteri*) in pigs with low-backfat thickness. Additionally, functional profiling of microbial communities indicated that the isoquinoline alkaloid biosynthesis, arachidonic acid metabolism, and styrene degradation were significantly different between low- and high-backfat thickness groups. Furthermore, feeding *L. reuteri* reduced the intake-to-gain ratio and had the potential to reduce backfat thickness. These findings show that microbiota could alter the production performance of pigs.

**Abstract:**

The backfat thickness of pigs not only affects the physical properties and taste of meat, but it also closely relates to the reproduction performance of sows. Accumulating evidence indicates that, apart from genetic factors, gut microbiota can also modulate the fat deposition and muscle growth. However, the differential microbiota in pigs with different backfat thickness, and whether microbiota affects backfat thickness, remains elusive. Firstly, 16S ribosomal RNA (16S rRNA) gene sequencing was performed on 62 fecal samples from pigs with different backfat thicknesses, and the compositions of microbiota among different groups with different backfat thicknesses were different. The abundance of *Lactobacillus. reuteri* (*L. reuteri*) and *Prevotella sp RS2* was significantly higher in pigs with low-backfat thickness than that in pigs with middle and high-backfat thickness; meanwhile, the abundance of *Desulfovibrio piger* was significantly lower (*p <* 0.05) in pigs with low-backfat thickness. Furthermore, the functional profiling of microbial communities suggested that the abundance of isoquinoline alkaloid biosynthesis and styrene degradation were significantly lower (*p <* 0.05) in the low-backfat thickness group than that in middle and high-backfat thickness groups. Finally, *L. reuteri* fed to Meishan piglets was capable of improving the production performance and had the potential to reduce backfat thickness. This study provides new evidence that microbiota can regulate the phenotype of the host, and dietary supplementation with *L. reuteri* can improve the production performance of piglets.

## 1. Introduction

The thickness of the backfat of pigs not only affects the physical and chemical properties of meat, but it is also closely associated with the reproductive performance of sows [1]. Due to the long-term breeding process, excessive attention has been paid to the selection of backfat thickness, lean meat percentage, and the growth rate, which seriously affect meat quality. On the one hand, excessive backfat thickness hinders the reproductive ability of sows [2,3]. On the other hand, during the stage of gestation, too-thin backfat thickness reduces the number of swine born alive. Thus, suitable and stable backfat thickness is essential for maintaining the reproductive performance of sows [4]. Importantly, fat deposition and muscle growth on the back are a very complex process. A large number of genes are involved in fat deposition and muscle growth [5,6]. Additionally, environmental factors, including nutrition and feeding management, also affect fat deposition.

It is currently believed that backfat thickness is related to genetic factors. Backfat thickness is a quantitative trait with economic value and has high heritability. Five hundred and ninety-four quantitative trait loci (QTL) were related to the average backfat thickness (https://www.animalgenome.org/cgi-bin/QTLdb/SS/qtrait?trait_ID=2, accessed on 26 February 2022) Additionally, some genes were reported to have the function of regulating backfat thickness. Insulin-like growth factor (*IGF2*) is one of the genes related to fat deposition, which is highly expressed in the liver, and plays a major role in cell proliferation, differentiation, transformation, and metastasis [7]. Additionally, the *IGF2* gene of pigs is related to the growth rate, the content of intramuscular fat, the thickness of backfat, and the eye muscle area [7]. Lysine demethylase 2A (*KDM2A*), which participates in cell-cycle transformation and DNA methylation, is linked to the average daily gain (ADG) of pigs from birth to market [7]. Furthermore, translation elongation factor 1 (*TEF1*) is highly expressed in skeletal muscle, is a critical transcription factor, and is TEF-1’s highly conserved subunit TAE can interact with the M-CAT element, and the latter of which regulates the expression of muscle-specific genes [8,9]. Genes for the ADG and backfat thickness of pigs are mainly located on chromosomes 1, 2, 4, and 7 [10].

Pig intestines are rich in microorganisms, which play an important role in the growth and development of the animal body. Intestinal microbiota, the largest symbiotic ecosystem within the host, can interact with the intestinal mucosa and maintain intestinal homeostasis. Importantly, intestinal microorganisms can be involved in the process of energy intake and carbohydrate metabolism. Certain intestinal microorganisms produce antibacterial substances, such as antibiotics, lipopeptides, and glucanase [11]. Additionally, microorganisms can also stimulate the body to produce defensins and interferons to resist the invasion of external pathogens [12,13]. Recent studies have shown that intestinal microbiota can regulate the fat deposition of the host. Firmicutes in mouse intestines can promote fat deposition [14]. Additionally, it was found that obese pigs have a lower diversity in gut microbiota than lean pigs, and the abundance of Firmicutes is lower than that of lean pigs. Microbiota depletion promotes the browning of white adipose tissue and reduces obesity, and it can also promote the development of functional beige fat in the inguinal subcutaneous adipose tissue and perigonadal visceral adipose tissue [15]. *L. reuteri* is a kind of probiotic that colonizes a variety of mammals, and the abundance of *L. reuteri* is different in different individuals [16]. Serval beneficial effects of *L. reuteri* has been reported. Firstly, *L. reuteri* can secrete some bioactive molecules. *L. reuteri* was reported to secrete reuterin to inhibit the growth of harmful bacteria, such as *Enterotoxigenic Escherichia coli* and *Salmonella* [17]. In addition, *L. reuteri* can also increase the content of butyric acid in the intestine which helps maintain intestinal homeostasis. Moreover, *L. reuteri*, as a lactic acid bacterium, is capable of producing lactic acid and a variety of enzymes in animal intestines, such as lipase and bile salt hydrolase, which are beneficial in improving the pH of animal intestines, inhibit bacterial growth, and improve feed utilization. Additionally, *L. reuteri* can synthesize B vitamins to enhance the growth performance of poultry and domestic animals [18]. Overall, *L. reuteri* plays a major part in the metabolism and the development of the intestine.

The Chinese Meishan pig breed is well-known for its high reproductive performance, great meat physical properties, and tolerance for rough feed. It is also known that Meishan pigs have a thicker backfat than commercial breeds, such as Landrace [19,20]. Hence, how to reduce the backfat thickness of Meishan pigs is one of the issues that the pig industry needs to address. Gut microbiota can regulate phenotypes, including the growth and development of mammals by different mechanisms. Herein, the purpose of this study is to explore the changes of fecal microbes from pigs with different backfat thicknesses by using 16S rRNA gene sequencing, and to further investigate the potential impact of microbiotas on the productive performance and backfat thickness.

## 2. Materials and Methods

### 2.1. Animal Management and Sample Collection

A total of 62 150-day-old crossbred pigs (Large White × Landrace, all gilts) with a similar weight (85 ± 2.0 kg) from a large-scale pig farm in Jiangsu Province were used for the sample collection. All pigs were kept on the same farm under standardized conditions with ad libitum access to water, and a commercial formula diet was provided to pigs two times a day. Pigs were randomly distributed to pens and there were 15–20 pigs per pen. All pigs were in healthy condition and had not been fed with antibiotics for at least 3 months prior to collecting fecal samples. Pigs were divided into three groups on the basis of backfat thickness (7–14 mm, *n* = 23; 14–20 mm, *n* = 23; 20–28 mm, *n* = 16). The backfat thickness of the pigs was measured by using an A-mode ultrasonography Lean-meater (Renco, Minneapolis, MN, USA) and measurements were taken by the same employee throughout the trial. The total measurement range and accuracy of this apparatus, including skin, was 4–35 mm, ±1 digit. The operating procedures were as follows: briefly, the P2 site (approximately 6–8 cm away from the dorsal midline at the last rib curve) was found, and the couplant was applied to the pigs after the excess hair was removed. Then, the probe was closely fitted to the measured site, and the backfat thicknesses of the pigs were measured based on the screen of the machine.

Fresh stool was collected in sterile tubes by stimulating the perianal area when pigs were 150-days old with an empty stomach, and the fresh stool was snap-frozen in liquid nitrogen, then stored at −80 °C until DNA extraction.

### 2.2. DNA Extraction and PCR Amplification

The fecal samples were thawed and ~300 mg fecal samples were utilized to extract the microbial genomic DNA by the QIAmp DNA Stool Mini Kit (Qiagen, Hilden, Germany). The DNA purity and yield were examined through 1% agarose gels. The V4 region of the 16S rRNA gene was chosen for the identification of bacterial species. The primer pair 341F/806R (341F: CCTACGGGNGGCWGCAG; 806R: GGACTACHVGGGTATCTAAT) was used to amplify the V4 region. PCR reactions were carried out in triplicate with a 50 μL mixture consisting of 5 μL of KOD buffer, 5 μL of 2 mM dNTP, 3 μL of 25 mM MgSO_4_, 1.5 μL of forward/reverse primers (10 mM), 1 μL of KOD Polymerase, and 100 ng of template DNA (Toyobo, Osaka, Japan). Barcoded V4 amplicons were sequenced on an Illumina HiSeq2500 platform (Illumina, San Diego, CA, USA) following the standard protocols.

### 2.3. 16. S Ribosomal RNA Sequencing and Analysis

#### 2.3.1. Quality Control and Reads Merge

Briefly, the primer, low-quality, and barcode sequences were removed to obtain clean sequence reads. FLASH (Version 1.2.11) [21] was used to merge the paired-end clean reads with a minimum overlap of 10 bp and mismatch error rates of 2%. The quality control details are shown in Supplementary Table S1.

The UPARSE (Version 9.2.64) [22] pipeline was used to cluster effective tags into operational taxonomic units (OTUs) of 97% similarity. We removed OTUs with a relative abundance of 0.01% and less than 1% presence in experimental pigs from further analysis. The tag sequence with the highest abundance was elected as the representative sequence within each cluster. Between groups, Venn analysis was performed in the R project’s VennDiagram package (Version 1.6.16) [23] and an UpSet plot was performed in the R project’s UpSetR package (Version 1.3.3) [24] to identify unique and common OTUs.

#### 2.3.2. Alpha and Beta Diversity Analysis

Chao1 and Shannon indices were computed by QIIME (Version 1.9.1) [25]. OTUs’ rarefaction curve was generated in the R project’s ggplot2 package (Version 2.2.1) [26]. Alpha index comparisons in different groups were calculated using the Kruskal–Wallis H test in the R project’s Vegan package (Version 2.5.3) [27].

The R project’s Vegan package (Version 2.5.3) [27], plotted in the R project’s ggplot2 package (Version 2.2.1) [26], was applicated to compute multivariate statistical techniques, including principal coordinates analysis (PCoA), non-metric multidimensional scaling (NMDS) of the weighted-UniFrac, and Bray–Curtis distances. The R project’s Vegan package was used to analyze the Kruskal–Wallis H test.

#### 2.3.3. Function Prediction

The microbial gene function was predicted using the PICRUSt software (Version 2.1.4) [28]. The predicted genes and their functions were then annotated using the Kyoto Encyclopedia of Genes and Genomes (KEGG) database. Welch’s test was used to calculate function differences between groups in the Vegan package of the R project (Version 2.5.3) [27].

### 2.4. Meishan Pigs Feeding Experiments

Eighteen 35-day-old female Meishan pigs of a similar weight were separated into two groups randomly (*n* = 9 per group) and kept under standardized conditions. All pigs had ad libitum access to water and were orally administrated with corn- or soy-based fodder, or *L. reuteri* (CICC 6118) plus basal fodder three times per day. The control group were fed with basal fodder for 35 days. The *L. reuteri* powder (5 × 10^10^ CFU/kg) evenly added into the basal fodder was fed to pigs for 10 days, and then changed to basal fodder for 25 days.

The body weight of the Meishan pigs with an empty stomach was recorded at day 35 and day 70, and the weight gain and the average daily gain (ADG) of pigs during the experiments were recorded. The backfat thickness at the back P2 point was recorded using an A-mode scanner Lean-meater (Renco, Minneapolis, MN, USA) at the end of the experiments. Additionally, the intake amount of the pigs was calculated based on the total amount of the fodder and the daily feed refusals each morning, and the average daily feed intake (ADFI) was calculated. The feed conversion rate was calculated by the ratio of ADFI-to-ADG. SPSS (Version 21.0) software (IBM Corp, Armonk, NY, USA) was used to carry out an independent sample t-test and a one-way analysis of variance (ANOVA), and the data are expressed as means ± standard deviation (Mean ± SD). * *p <* 0.05. NS, not significance.

## 3. Results

### 3.1. The Richness of Fecal Microbes in Pigs with Different Backfat Thickness

**Figure 1:**
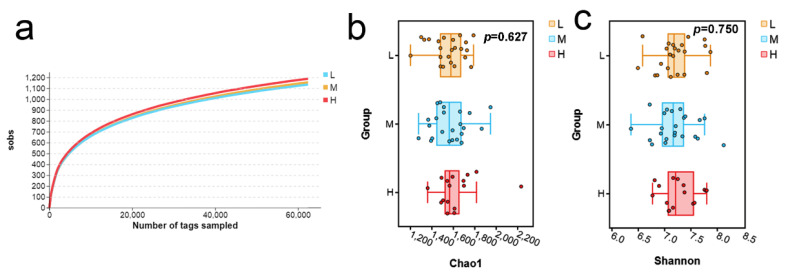
Alpha-diversity analysis of fecal microbes in each group. (**a**) Rarefaction curves. (**b**,**c**) Boxplot based on the Chao1 and Shannon index.

The backfat thickness was measured from different pigs by using the A-mode ultrasonography. The use of L, M, and H represent low- (7–14 mm, *n* = 23), middle- (14–20 mm, *n* = 23), and high-backfat thicknesses (20–28 mm, *n* = 16), respectively. The 16S rRNA sequencing generated 6600919 clean reads and 6574015 clean tags from 62 samples (Supplementary Table S1). The rarefaction curve was performed to compare species richness in different groups, which suggested the richness was similar among different groups (Figure 1a). In order to further detect the richness of fecal microbes in pigs with different backfat thicknesses, the Chao1 index and the Shannon index were used to reflect the alpha diversity in separate groups. The Chao1 index of the fecal microbes of the low-, middle-, and high-backfat thickness groups was 1555.09, 1568.76, and 1629.25, respectively. In addition, the Shannon index showed the diversity of fecal microbes with different backfat thicknesses, which was 7.22, 7.20, and 7.28, respectively. Moreover, the Kruskal–Wallis (KW) rank sum test was performed based on the Chao1 and Shannon index to test the significance, and both the Chao1 index (*p =* 0.627) and the Shannon index (*p =* 0.750) did not exhibit a significant difference among different groups (Figure 1b,c).

### 3.2. Analysis of Microbial Community and Structure Composition

**Figure 2:**
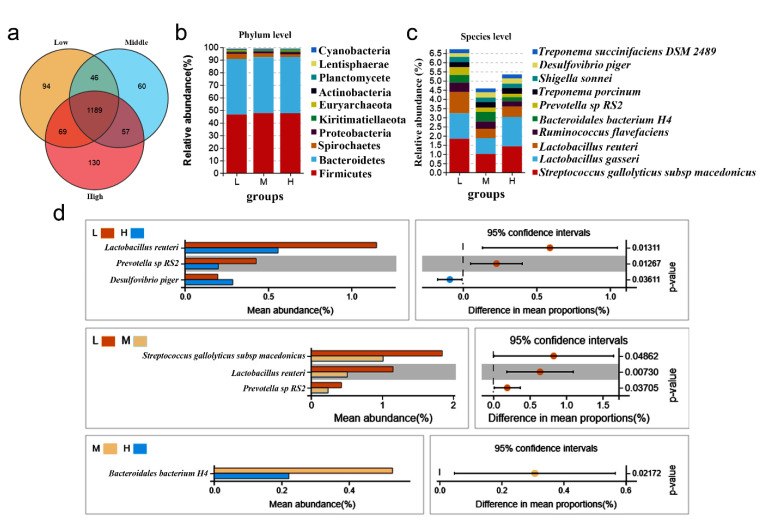
Microbial composition and diversity among the groups with different backfat thickness. (**a**) Venn plot of OTU numbers in each group with different backfat thickness. (**b**) Summary of bacterial phylum detected in the three groups. (**c**) Summary of bacterial species detected in the three groups. (**d**) Significance analysis of species abundance between different backfat thickness groups based on the Welch’s *t*-test.

A cluster analysis of OTUs was carried out to explore the structure and composition of different groups. Supplementary Table S2 lists the details of tags and OTUs from different samples. The Venn chart shows that there were 1189 shared OTUs in pig feces with different backfat thicknesses, accounting for 80% of all OTUs; there were 94 unique OTUs for the low-backfat thickness group, 60 unique OTUs for the middle-backfat thickness group, and 130 unique OTUs for the high-backfat thickness group (Figure 2a), which suggests that the compositions among different groups were different and there was a potential relationship between the phenotype of backfat thickness and microbiotas. OTU analysis also revealed that, at the phylum level, the Bacteroides and Firmicutes accounted for the highest proportions in each group, and even exceeded 90% (Figure 2b). At the species level, *Streptococcus, Lactobacillus gasseri, Lactobacillus reuteri, Ruminococcus flavefaciens, Bacteroidales bacterium H4, and Prevotella sp RS2* differed in composition among the three groups (Figure 2c). In order to further explore the differences in the bacteria species of pigs among different groups, Welch’s t-test was computed. The results showed that the abundance of *L. reuteri* was significantly higher in the low-backfat thickness group than that in the high-backfat thickness group (*p =* 0.013) and in the middle-backfat thickness group (*p =* 0.007) (Figure 2d). *Prevotella sp RS2* (*p* *=* 0.013) also had a higher abundance in the low-backfat thickness group compared to that in the high-backfat thickness group. Otherwise, *Desulfovibrio piger* (*p =* 0.036) had a lower abundance in the low-backfat thickness group than that in the high-backfat thickness group. Additionally, the abundance of *Bacteroidetes bacterium H4* was higher in the middle-backfat thickness group compared to that in the high-backfat thickness group. These results indicate that the microbial community and structure compositions among groups with different backfat thickness were different, and *L. reuteri* may take part in regulating backfat thickness.

### 3.3. Community Structures of Different Backfat Thickness Groups

**Figure 3:**
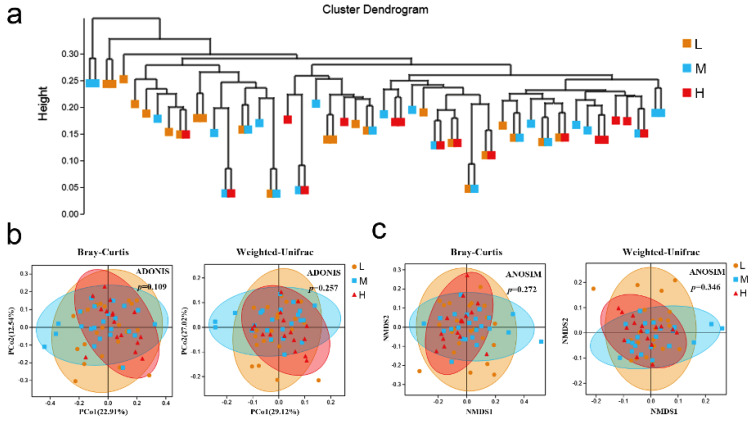
Beta-diversity analysis of fecal microbes among different groups. (**a**) UPGMA cluster tree was used to show the similarity among different groups. (**b**) PCoA based on the Bray–Curtis dissimilarities or weighted-UniFrac dissimilarities. (**c**) NMDS built on the Bray–Curtis dissimilarities or the weighted-UniFrac dissimilarities.

Beta diversity was analyzed to reflect the difference among different groups. The results constructed by an unweighted pair group method with arithmetic mean (UPGMA) algorithm reflected the similarities and differences between multiple samples. UPGMA clustering of microbiome taxonomic profiles among groups using the Bray distance did not show a clear separation among the three groups (Figure 3a). In addition, PCoA based on the Bray–Curtis distance and weighted-UniFrac distance, respectively, was performed, and the results showed that the samples did not obviously cluster based on neither the Bray–Curtis distance (ADONIS; L vs. M vs. H, *p =* 0.109), nor the weighted-UniFrac distance (ADONIS, L vs. M vs. H, *p =* 0.257) (Figure 3b).

In order to show the non-linear structural relationship of the ecological data between the samples, in this study, we used NMDS to display the species information contained in the samples in the form of points in a multi-dimensional space. The results indicated that there was no significant separation between samples from different groups based on neither the Bray distance (ANOSIM; L vs. M vs. H, *p =* 0.272), nor the weighted-UniFrac distance (ANOSIM; L vs. M vs. H, *p =* 0.346) (Figure 3c).

### 3.4. Functional Profiling of Microbial Communities in Different Backfat Thickness Groups

**Figure 4:**
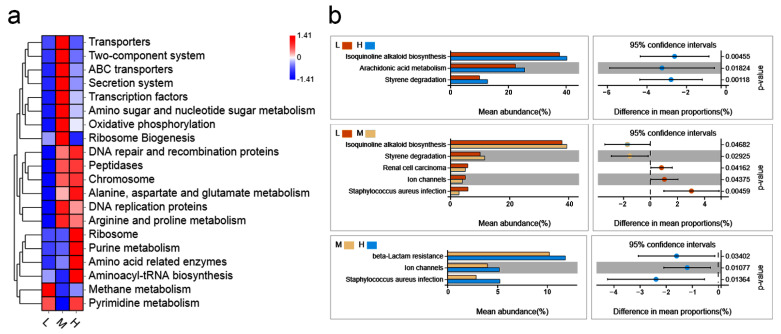
Functional analysis of microbes. PICRUSt software was used to analyze the function of microbes among different groups. (**a**) Heat map based on the abundance of the top 20 functional pathway. (**b**) Significance analysis of functional pathway based on the Welch’s t-test.

PICRUSt software was utilized to investigate the function of microbiota given the 16S rRNA sequencing genome [28]. In order to further research the differences in the KEGG pathways among diverse groups, the function distribution heat map of all groups was drawn. The results showed that the abundance in the metabolic pathways was high in all three backfat thickness groups (Supplementary Table S3), which suggests that microbiotas regulate the host phenotype mainly by affecting the process of metabolism. Additionally, the signaling pathways in the low-backfat thickness group were enriched in methane metabolism and pyrimidine metabolism. The high-backfat thickness group had a higher relative abundance in ribosomes, purine metabolism, amino-acid-related enzymes, and aminoacyl-tRNA biosynthesis functional pathways (Figure 4a and Supplementary Table S4). As Figure 4b shows, the isoquinoline alkaloid biosynthesis (*p =* 0.0046), arachidonic acid metabolism (*p =* 0.0182), and styrene degradation (*p =* 0.0118) of the functional pathway was exhibited significantly less in the low-backfat thickness group compared with that in the high-backfat thickness group. Additionally, the abundance of isoquinoline alkaloid biosynthesis (*p =* 0.0468) and styrene degradation (*p =* 0.0293) was also significantly lower in the low-backfat thickness group than that in the middle-backfat thickness group (Figure 4b), suggesting that the isoquinoline alkaloid biosynthesis and styrene degradation influenced by microbiotas plays an important role in backfat thickness. Additionally, the abundance of beta-lactam resistance, ion channels, and staphylococcus aureus infection was significantly lower in the middle-backfat thickness group than that in the high-backfat thickness group (Figure 4b). Overall, these results suggest that microbiotas may influence backfat thickness by regulating the functions of isoquinoline alkaloid biosynthesis and styrene degradation.

### 3.5. Feeding L. reuteri Improved Piglets Production Performance

**Figure 5:**
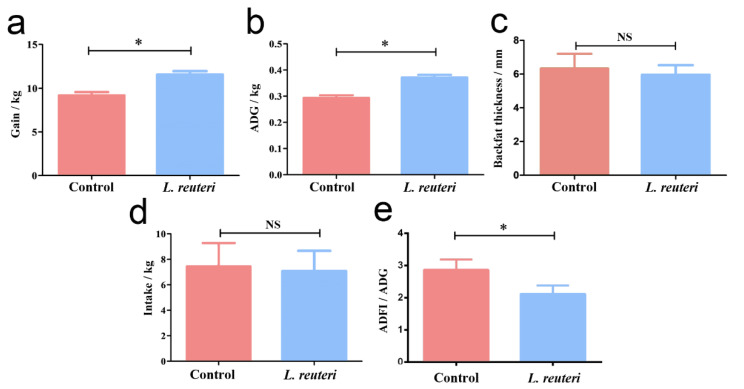
Feeding *L. reuteri* improved production performance of piglets. (**a**) Gain of piglets from original to the final. (**b**) Average daily gain of piglets. (**c**) The backfat thickness of piglets (**d**) Intake of piglets. (**e**) The intake to gain ratio, * *p <* 0.05 significant differences between treatments. NS, not significance differences (*p* > 0.05).

The results described above indicate that the abundance of *L. reuteri* in the low-backfat thickness group is greater than that of the high-backfat thickness group, hinting at a potential role that *L. reuteri* plays in the phenotype of backfat thickness. The Meishan pig breed has thicker backfat than commercial breeds. In order to further investigate whether *L. reuteri* also has the same effect on Meishan pigs with a thicker backfat thickness, the animal experiment was constructed and the Meishan piglets were continuously fed with *L. reuteri* for 10 days. The results showed that the weight gain of pigs fed with *L. reuteri* was significantly higher than that of the control group in the entire state under the same management condition (*p <* 0.05) (Figure 5a). Moreover, the average daily gain (ADG) during the experiments was also significantly higher than that of the control group (*p <* 0.05) (Figure 5b). Importantly, the backfat thickness of the control group was 6.33 mm, whereas the backfat thickness of the treated group was 5.96 mm, suggesting that feeding Meishan piglets with *L. reuteri* for only 10 days could reduce their backfat thickness (*p >* 0.05) (Figure 5c). Meanwhile, to detect the feeding utilization rate influenced by *L. reuteri*, the feed-to-gain ratio was calculated and compared between the two groups. Notably, no obvious difference was observed in the intake between the two groups (*p >* 0.05) (Figure 5d). The feed conversion rate was calculated by the ratio of average daily feed intake-to-average daily gain, and the results showed that the ratio of feed-to-meat in the *L. reuteri*-treated group was significantly lower than that of the untreated group (*p <* 0.05) (Figure 5e), indicating that feeding Meishan piglets with *L. reuteri* could improve their feed utilization and production performance.

## 4. Discussion

Backfat thickness is an important trait which reflects the body condition of pigs. The backfat thickness of sows before farrowing has a more significant impact on the birth weight and the number of piglets born alive [1]. Studies have shown that the optimal backfat thickness range of sows is different at different stages [29,30]. The sow’s backfat thickness should not be less than 15 mm during breeding, and the optimal backfat thickness gradually increases with the development of gestation, but the highest backfat thickness should not be greater than 22 mm. Additionally, backfat thickness influences sow’s lactation, over-thick or over-thin backfat thickness impairs the lactation of sows and hinders the uterus function [31]. Hence, maintaining suitable and stabled backfat thickness throughout the reproductive cycle is more important than fixing this parameter by breeding alone. Thus, it is essential to maintain a moderate backfat thickness and explore the factors which affect backfat thickness. However, the relationship between microbiota and backfat thickness, and how microbiota regulates the backfat thickness and production performance of pigs, remains unknown. This study found that an abundance of *L. reuteri* was higher in pigs with low-backfat thickness, and dietary supplementation of *L. reuteri* has the potential to reduce the backfat thickness and improve the performance of Meishan piglets, which provides new evidence that the feeding of probiotics is beneficial for the growth and development of animals.

The gut microbiota has the capability to change the phenotypes of the host; intriguingly, it confers the host the ability to consume intractable food sources, which opens a new ecological possibility to the host. Accumulating evidence suggests that the gut microbiota is linked to many different modern-day illnesses, such as obesity [32,33] and diabetes [34]. Studies in humans and mice have demonstrated that, in obese individuals, the Firmicutes had a larger proportion [35], and Bacteroides had a lower proportion [36]. The abundance of *Bactericide thetaiotaomicron*, which can secrete short-chain fatty acids to inhibit the accumulation of excess fat and prevent obesity, was reduced in obese individuals [32]. Additionally, the obesity-associated microbiota alters the host’s energy harvesting and fat deposition. Intestinal microbiota can regulate central appetite and food-reward signaling, which together have crucial roles in obesity [37]. Pigs are considered to be biomedical models for energy metabolism and obesity in humans because of their similar metabolic features. In this study, among the groups with different backfat thicknesses, the proportions of Firmicutes and Bacteroides were in higher abundance. Elevated levels of Firmicutes and depleted levels of Bacteroidetes were identified in obese individuals [38]. Moreover, all three groups with different backfat thicknesses had 1189 shared OTUs: the difference was that there were 94 unique OTUs in the low-backfat thickness group, 60 unique OTUs in the middle-backfat thickness group, and 130 unique OTUs in the high-backfat thickness group, which suggested that the compositions and the structures of microbiota from pigs with different backfat thickness were distinct. These results prompted the notion that there may be one or some certain bacteria that could affect the fat deposition and further influence the growth and development of pigs. In line with previous studies which indicated that fatness-associated OTUs were mainly ascribed to *Prevotella* [39], the abundance of *Prevotella sp RS2* in the low-backfat thickness group was much higher than that of the middle-backfat thickness group and the high-backfat thickness group, suggesting that *Prevotella* is able to regulate lipid metabolism. In this study, importantly, we also found that, compared with the high-backfat thickness group and aside from *Prevotella sp RS2*, the abundance of *L. reuteri* was also higher in the low- and middle-backfat thickness group, hinting that *L. reuteri* may participate in the regulation of the fat deposition of the back. Jiang et al. indicates that *Lactobacillus reuteri A9* and *Lactobacillus mucosae A13* reduced total cholesterol levels [40]. *Lactobacillus reuteri HI120* also has the potential to reduce serum cholesterol levels in obese mice [41]. A recent study also reported that, in obese mice models, *Lactobacillus reuteri J1* changes gut microbiota to prevent obesity by regulating bile acid metabolism [42]. Taken together, these results suggest that *Prevotella* and *L. reuteri* may play essential roles in the fat deposition of backfat by regulating lipid metabolism, and they have the potential to prevent obesity.

The Meishan pig breed is a Chinese indigenous pig breed which is well known for its precocious and prolific traits [43,44]. Nevertheless, the Chinese Meishan pig breed has a higher backfat thickness than that of other commercial pig breeds, such as Landrace [19,20], and the too-high backfat thickness of the Meishan pig breed is an issue that affects the higher lean-meat percentage [45]. Hence, reducing the backfat thickness of the Meishan pig breed by a convenient method, such as feeding probiotics, is an effective way to change this phenotype. Weaning at around 28 to 35 days old is a stressful event which impairs the function of the intestinal and immune systems and further results in the reduced production performance of piglets [46,47]. Furthermore, the nursing phase of piglets after weaning has a profound influence on the growth and development of pigs. Hence, the feeding management of nursing pigs is very vital to the pig industry. In this study, we found that *L. reuteri* with a higher abundance in crossbred gilts with low-backfat thickness can also influence the production performance of Meishan piglets. Feeding piglets with *L. reuteri* significantly increased the average daily gain and reduced the feed-to-gain ratio of piglets. In accordance with the study in which oral administration of *L. reuteri* D8 significantly increased the body weight and improved the development of intestines of 3-day-old Meishan piglets [48], the increased mucosal, villi length, lipase, and protease may contribute to the improved production performance of Meishan piglets [49]. Additionally, *L. reuteri* fed to piglets reduced backfat thickness by 0.37 mm on average, although it did not reach the significant level. This may be due to the short feeding period, suggesting that feeding *L. reuteri* has the potential to reduce the backfat thickness of Meishan pigs. Moreover, experiments with a longer feeding time and more pigs will be needed to further determine the effect of *L. reuteri* on fat deposition. Studies report that some metabolites produced by *L. reuteri* can participate in the regulation of the pathways in lipid metabolism, including the AMPK signaling pathway. The short-chain fatty acids produced by *L. reuteri* may reduce backfat thickness of Meishan piglets by regulating lipid metabolism [50,51,52]. Collectively, feeding *L. reuteri* to piglets could alter the metabolism process and improve feed utilization.

## 5. Conclusions

Taken together, in this study, we found that *L. reuteri* has a higher abundance of fecal microbiota from pigs with low-backfat thickness, and the biological functions of microbiota is mainly associated with the metabolism pathway. Importantly, feeding *L. reuteri* to Meishan pigs could enhance their average daily gain and decrease their feed to gain ratio.

## Data Availability

Data are contained within the article or supplementary material, and the 16S rRNA gene sequencing data has been uploaded to the SRA database with the accession number PRJNA705538.

## Supplementary Materials

The following are available online at https://www.mdpi.com/article/10.3390/vetsci9100527/s1, Table S1: Information of clean tags; Table S2: Details of tags and OTUs; Table S3: KEGG orthology analysis; Table S4: KEGG pathway analysis.

## References

[1] Amaral Filha W.S., Bernardi M.L., Wentz I., Bortolozzo F.P. (2010). Reproductive performance of gilts according to growth rate and backfat thickness at mating. Anim. Reprod. Sci..

[2] Zhou Y., Xu T., Cai A., Wu Y., Wei H., Jiang S., Peng J. (2018). Excessive backfat of sows at 109 d of gestation induces lipotoxic placental environment and is associated with declining reproductive performance. J. Anim. Sci..

[3] Tian L., Huang J., Wen A., Yan P. (2020). Impaired mitochondrial function results from oxidative stress in the full-term placenta of sows with excessive back-fat. Animals.

[4] Amdi C., Giblin L., Ryan T., Stickland N.C., Lawlor P.G. (2014). Maternal backfat depth in gestating sows has a greater influence on offspring growth and carcass lean yield than maternal feed allocation during gestation. Anim. Int. J. Anim. Biosci..

[5] Wang H., Wang J., Yang D.D., Liu Z.L., Zeng Y.Q., Chen W. (2020). Expression of lipid metabolism genes provides new insights into intramuscular fat deposition in Laiwu pigs. Asian-Australas J. Anim. Sci..

[6] Mu X., Cui X., Liu R., Li Q., Zheng M., Zhao G., Ge C., Wen J., Hu Y., Cui H. (2019). Identification of differentially expressed genes and pathways for abdominal fat deposition in ovariectomized and sham-operated chickens. Genes.

[7] Han X., Yang H., Jiang T., Zhang Q., Zeng C., Fan B., Liu B. (2014). Investigation of four candidate genes (IGF2, JHDM1A, COPB1 and TEF1) for growth rate and backfat thickness traits on SSC2q in Large White pigs. Mol. Biol. Rep..

[8] Wen C., Yan W., Sun C., Ji C., Zhou Q., Zhang D., Zheng J., Yang N. (2019). The gut microbiota is largely independent of host genetics in regulating fat deposition in chickens. Isme J..

[9] Chauhan V., Kanwar S.S. (2021). Lipopeptide(s) associated with human microbiome as potent cancer drug. Semin. Cancer Biol..

[10] Sanchez M.-P., Riquet J., Iannuccelli N., Gogué J., Billon Y., Demeure O., Caritez J.-C., Burgaud G., Fève K., Bonnet M. (2006). Effects of quantitative trait loci on chromosomes 1, 2, 4, and 7 on growth, carcass, and meat quality traits in backcross Meishan × Large White pigs1. J. Anim. Sci..

[11] Niu Q., Zhang G., Zhang L., Ma Y., Shi Q., Fu W. (2016). Purification and characterization of a thermophilic 1,3-1,4-β-glucanase from *Bacillus methylotrophicus* S2 isolated from booklice. J. Biosci. Bioeng..

[12] Meade K.G., O’Farrelly C. (2018). β-Defensins: Farming the Microbiome for Homeostasis and Health. Front. Immunol..

[13] Martin P.K., Cadwell K. (2020). Regulation of interferon signaling in response to gut microbes by autophagy. Gut Microbes.

[14] Zhang Y., Liu Y., Li J., Xing T., Jiang Y., Zhang L., Gao F. (2020). Dietary corn-resistant starch suppresses broiler abdominal fat deposition associated with the reduced cecal Firmicutes. Poult. Sci..

[15] Suárez-Zamorano N., Fabbiano S., Chevalier C., Stojanović O., Colin D.J., Stevanović A., Veyrat-Durebex C., Tarallo V., Rigo D., Germain S. (2015). Microbiota depletion promotes browning of white adipose tissue and reduces obesity. Nat. Med..

[16] Mu Q., Tavella V.J., Luo X.M. (2018). Role of *Lactobacillus reuteri* in human health and diseases. Front. Microbiol..

[17] Sung H.W., Chen C.N., Liang H.F., Hong M.H. (2003). A natural compound (reuterin) produced by *Lactobacillus reuteri* for biological-tissue fixation. Biomaterials.

[18] Taranto M.P., Vera J.L., Hugenholtz J., De Valdez G.F., Sesma F. (2003). *Lactobacillus reuteri* CRL1098 produces cobalamin. J. Bacteriol..

[19] White B.R., Lan Y.H., McKeith F.K., Novakofski J., Wheeler M.B., McLaren D.G. (1995). Growth and body composition of Meishan and Yorkshire barrows and gilts. J. Anim. Sci..

[20] Legault C. (1985). Selection of breeds, strains and individual pigs for prolificacy. J. Reprod. Fertil. Suppl..

[21] Magoč T., Salzberg S.L. (2011). FLASH: Fast length adjustment of short reads to improve genome assemblies. Bioinformatics.

[22] Edgar R.C. (2013). UPARSE: Highly accurate OTU sequences from microbial amplicon reads. Nat. Methods.

[23] Chen H., Boutros P.C. (2011). VennDiagram: A package for the generation of highly-customizable Venn and Euler diagrams in R. BMC Bioinform..

[24] Conway J.R., Lex A., Gehlenborg N. (2017). UpSetR: An R package for the visualization of intersecting sets and their properties. Bioinformatics.

[25] Caporaso J.G., Kuczynski J., Stombaugh J., Bittinger K., Bushman F.D., Costello E.K., Fierer N., Peña A.G., Goodrich J.K., Gordon J.I. (2010). QIIME allows analysis of high-throughput community sequencing data. Nat. Methods.

[26] Wickham H., Chang W. Ggplot2: An Implementation of the Grammar of Graphics. Rpackage Version 0.7. https://www.semanticscholar.org/paper/ggplot%3A-An-implementation-of-the-Grammar-of-in-R-Wickham/7f3e2207d2ef8fc0cee74069879c8adf35303a91.

[27] Oksanen J., Kindt R., Legendre P., O’Hara B., Stevens H., Oksanen M. (2010). The Vegan Package. Vegan: Community Ecology Package. https://cran.r-project.org/web/packages/vegan/index.html.

[28] Langille M.G., Zaneveld J., Caporaso J.G., McDonald D., Knights D., Reyes J.A., Clemente J.C., Burkepile D.E., Vega Thurber R.L., Knight R. (2013). Predictive functional profiling of microbial communities using 16S rRNA marker gene sequences. Nat. Biotechnol..

[29] Tummaruk P., Sumransap P., Jiebna N. (2014). Fat and whey supplementation influence milk composition, backfat loss, and reproductive performance in lactating sows. Trop. Anim. Health Prod..

[30] Cheng C., Wu X., Zhang X., Zhang X., Peng J. (2019). Obesity of sows at late pregnancy aggravates metabolic disorder of perinatal sows and affects performance and intestinal health of piglets. Animals.

[31] De Rensis F., Gherpelli M., Superchi P., Kirkwood R.N. (2005). Relationships between backfat depth and plasma leptin during lactation and sow reproductive performance after weaning. Anim. Reprod. Sci..

[32] Liu R., Hong J., Xu X., Feng Q., Zhang D., Gu Y., Shi J., Zhao S., Liu W., Wang X. (2017). Gut microbiome and serum metabolome alterations in obesity and after weight-loss intervention. Nat. Med..

[33] Liu Y., Yin X.M., Xia R.W., Huo Y.J., Zhu G.Q., Wu S.L., Bao W.B. (2015). Association between the MUC4 g.243A > G polymorphism and immune and production traits in Large White pigs. Turk. J. Vet. Anim. Sci..

[34] Muñoz-Garach A., Diaz-Perdigones C., Tinahones F.J. (2016). Gut microbiota and type 2 diabetes mellitus. Endocrinol. Y Nutr. Organo De La Soc. Esp. De Endocrinol. Y Nutr..

[35] John G.K., Mullin G.E. (2016). The Gut Microbiome and Obesity. Curr. Oncol. Rep..

[36] Ley R.E., Turnbaugh P.J., Klein S., Gordon J.I. (2006). Microbial ecology: Human gut microbes associated with obesity. Nature.

[37] Torres-Fuentes C., Schellekens H., Dinan T.G., Cryan J.F. (2017). The microbiota–gut–brain axis in obesity. Lancet Gastroenterol. Hepatol..

[38] Riva A., Borgo F., Lassandro C., Verduci E., Morace G., Borghi E., Berry D. (2017). Pediatric obesity is associated with an altered gut microbiota and discordant shifts in *Firmicutes* populations. Environ. Microbiol..

[39] He M., Fang S., Huang X., Zhao Y., Ke S., Yang H., Li Z., Gao J., Chen C., Huang L. (2016). Evaluating the Contribution of Gut Microbiota to the Variation of Porcine Fatness with the Cecum and Fecal Samples. Front. Microbiol..

[40] Jiang J., Feng N., Zhang C., Liu F., Zhao J., Zhang H., Zhai Q., Chen W.J.F.M.L. (2019). *Lactobacillus reuteri* A9 and *Lactobacillus mucosae* A13 isolated from Chinese superlongevity people modulate lipid metabolism in a hypercholesterolemia rat model. FEMS Microbiol. Lett..

[41] Sun Y., Tang Y., Hou X., Wang H., Huang L., Wen J., Niu H., Zeng W., Bai Y.J.F.i.V.S. (2020). Novel *Lactobacillus reuteri* HI120 affects lipid metabolism in C57BL/6 obese mice. Front. Vet. Sci..

[42] Zhang C., Fang R., Lu X., Zhang Y., Yang M., Su Y., Jiang Y., Man C. (2022). *Lactobacillus reuteri* J1 prevents obesity by altering the gut microbiota and regulating bile acid metabolism in obese mice. Food Funct..

[43] Haley C.S., Lee G.J. (1993). Genetic basis of prolificacy in Meishan pigs. J. Reprod. Fertility. Suppl..

[44] Rothschild M., Jacobson C., Vaske D., Tuggle C., Wang L., Short T., Eckardt G., Sasaki S., Vincent A., McLaren D. (1996). The estrogen receptor locus is associated with a major gene influencing litter size in pigs. Proc. Natl. Acad. Sci. USA.

[45] Hoa V.B., Seo H.W., Seong P.N., Cho S.H., Kang S.M., Kim Y.S., Moon S.S., Choi Y.M., Kim J.H., Seol K.H. (2021). Back-fat thickness as a primary index reflecting the yield and overall acceptance of pork meat. Anim. Sci. J..

[46] Montagne L., Boudry G., Favier C., Le Huërou-Luron I., Lalles J.-P., Seve B. (2007). Main intestinal markers associated with the changes in gut architecture and function in piglets after weaning. Br. J. Nutr..

[47] Zhou Z., Zhang J., Zhang X., Mo S., Tan X., Wang L., Li J., Li Y., Ding X., Liu X. (2019). The production of short chain fatty acid and colonic development in weaning piglets. J. Anim. Physiol. Anim. Nutr..

[48] Wang M., Wu H., Lu L., Jiang L., Yu Q. (2020). Lactobacillus reuteri promotes intestinal development and regulates mucosal immune function in newborn piglets. Front. Vet. Sci..

[49] Dawood M.A., Magouz F.I., Salem M.F., Elbialy Z.I., Abdel-Daim H.A. (2020). Synergetic effects of Lactobacillus plantarum and β-glucan on digestive enzyme activity, intestinal morphology, growth, fatty acid, and glucose-related gene expression of genetically improved farmed tilapia. Probiotics Antimicrob. Proteins.

[50] Shen Y.L., Zhang L.Q., Yang Y., Yin B.C., Ye B.C., Zhou Y. (2022). Advances in the role and mechanism of lactic acid bacteria in treating obesity. Food Bioeng..

[51] Ni C., Li X., Wang L., Li X., Zhao J., Zhang H., Wang G., Chen W. (2021). Lactic acid bacteria strains relieve hyperuricaemia by suppressing xanthine oxidase activity via a short-chain fatty acid-dependent mechanism. Food Funct..

[52] Lew L.-C., Hor Y.-Y., Jaafar M.-H., Lau A.-S.-Y., Lee B.-K., Chuah L.-O., Yap K.-P., Azlan A., Azzam G., Choi S.-B. (2020). Lactobacillus strains alleviated hyperlipidemia and liver steatosis in aging rats via activation of AMPK. Int. J. Mol. Sci..

